# Acute Effects of Passive Stretching with and Without Vibration on Hip Range of Motion, Temperature, and Stiffness Parameters in Male Elite Athletes

**DOI:** 10.3390/jfmk10010017

**Published:** 2025-01-02

**Authors:** Daniel Jochum, Viola Vogel, Konstantin Warneke

**Affiliations:** 1 Department of Health Sciences and Technology, ETH Zurich, 8092 Zurich, Switzerland; daniel.jochum@hest.ethz.ch (D.J.); viola.vogel@hest.ethz.ch (V.V.); 2 Institute of Human Movement Science, Sport and Health, University of Graz, 8010 Graz, Austria

**Keywords:** stretching, vibratory stimulation, passive properties, flexibility, whole-body vibration

## Abstract

**Objectives**: Increasing exercise intensity and performance output with superimposed vibration gains interest, especially in high-performance training. However, the additional benefit of vibration in passive stretching exercises and its mechanisms remain unclarified. **Methods**: Passive stretching with (ST+V) and without (ST) vibration (20 Hz) was performed in male Olympic youth skiing athletes (*n* = 8, age: 17.9 ± 1.0 years) using a single-blinded randomized cross-over design. Acute hip abduction, hip anteversion, knee extension, and hamstrings (stand and reach straight leg raise) range of motion (ROM) were assessed using a digital goniometer, while stiffness was examined via MyotonPRO. The skin temperature of the whole leg was captured with infrared thermography and analyzed in different segments. **Results**: Both stretching interventions increased ROM compared to the control group (CG) (*p* < 0.001–0.033, d = 1.0–1.6) without differences between ST+V and ST (*p* = 0.202–0.999). While skin temperature decreased in the CG and ST, ST+V maintained a constant temperature in the lower legs. Stiffness was not affected by both stretching interventions. **Conclusions**: The stretching intervention leads to significant increases in flexibility, while additional vibration did not further enhance the ROM.

## 1. Introduction

When aiming to enhance joint range of motion (ROM), stretching is probably the most common intervention showing significant chronic, meaning long-term [1], and acute or immediate [2] increases. Explanatory approaches for acute measured effects are twofold. Most authors prefer a more neuronal approach and refer to changes in passive peak torque measured in the end ROM [3] as a determinant of the highest stretch torque subjects can tolerate [4]. In contrast, among others, reduced friction and stiffness [5,6] could also contribute to enhanced ROM [7]. Accordingly, Konrad et al. [8] found 2–5 minutes of (either static, dynamic, or ballistic) stretching to induce a sufficient stimulus to increase ankle dorsiflexion ROM with 3.5–4.5%, which was accompanied by muscle stiffness (−13.1–20.3%) and muscle-tendon stiffness (−10.5–13.7%) reductions in the gastrocnemius medialis measured with ultrasonography. Additionally, using 5 × 60 s stretching exercises reduced stiffness by about 25% [9]. Accordingly, Takeuchi et al. [10] reviewed the available literature and reported acute muscle-tendon unit stiffness reduction due to static stretching.

While stretching is effective, there are speculations that superimposing additional contractions of the muscle could accumulate effects. One possibility is to stimulate reflex mechanisms to induce contractions via vibrational stimuli with the so-called tonic vibration reflex (TVR) [11]. These could, among others, induce circulatory and thermoregulatory effects [12], thus, heat generation in the intervened muscle (exothermal reaction) [13]. In turn, this could also result in stiffness reduction, thixotropic effects, and improved ROM. Accordingly, previous studies confirmed that whole-body and local vibration stimuli provided a potent stimulus to increase ROM acutely [14,15].

However, current evidence often stems from performing superimposed vibration as opposed to a passive control condition. To attribute acute ROM increases to vibration stimulation, acute increases in ROM induced by general warm-up effects cannot be excluded [16]. Since many results stem from passively controlled intervention effects, conclusions based on such investigations seem questionable—as a result, they cannot be attributed solely to vibration when combined with exercises [15]. When investigating the relevance of vibration effects, it is necessary to oppose a stretching + vibration group to a stretching alone + a passive control condition [17]. This was conducted previously, while several studies found an additional effect [18,19], while others did not [17,20,21]. Most of these studies did not measure any underlying mechanism that might explain or facilitate ROM increases [15]. Only Rodrigues et al. [17] investigated a form of passive torque, which decreased only with conventional stretching.

Consequently, this study superimposed vibration on a stretching intervention and compared flexibility increases in different lower-leg muscles to a stretching-alone (active control) and a passive control condition. To increase homogeneity, we aimed to incorporate youth male elite athletes from the same team, which provide an already high ROM due to consistent flexibility routines. Therefore, we assumed the potential benefits of adding vibration to further increase the already high flexibility status. To explain the variance, leg muscle-tendon stiffness, as suggested as a moderator for ROM increases [10], as well as leg skin temperature changes, a contactless measurement technique to investigate and compare the magnitude of warm-up-related muscle temperature increases to facilitate ROM [22] was additionally evaluated. As suggested in previous articles, it was hypothesized that (a) vibration would further increase ROM compared to the active control, while (b) both stretching + vibration and stretching alone resulted in significant ROM increases and stiffness decreases. Further, (c) we expect vibration to improve ROM increases by elevating the muscle and skin temperature.

## 2. Materials and Methods

### 2.1. Experimental Approach

An assessor-blinded repeated measure within-subject factor study with a cross-over design was used, incorporating three sessions for each participant with an ensured wash-out period of 7 days. Participants were allocated by concealed lottery to a randomized sequence of the interventions stretching + vibration (ST+V) and without vibration (ST) condition (active control condition) or resting in a sitting position, which served as the passive control condition (CG). All participants were treated as initially allocated, and all interventions were performed by the same researchers at the same time of day to avoid the effect of circadian rhythms on the study results.

**Figure 1:**
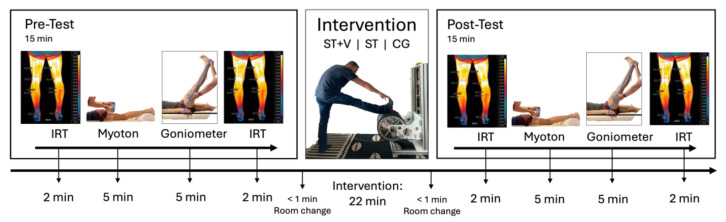
Time sequence of measurement procedure: Skin temperature with infrared thermography (IRT), muscle and tendon stiffness with myotonometry (Myoton), and range of motion (Goniometer) were tested before and immediately after one of three interventions (stretching training + vibration (ST+V), stretching training (ST), seated-rest control (CG)) without warm-up in a temperature-controlled room. Between measurement and intervention, participants had to change the room with a walk of around 25 m.

In the pre- and post-test, muscle and tendon stiffness, flexibility, and skin temperature were tested in the lower extremity (Figure 1). Legs were chosen for the investigation as several standardized ROM measurements exist [23] and as they are easy to access for palpable muscles and tendons for myotonometry and to areas for infrared thermography (IRT).

### 2.2. Participants

Male elite sport athletes from the youth skiing team “NLZ West” (Olympic center in Oberstdorf, Germany) (*n* = 8, age: 17.9 ± 1.0 years, body weight: 82.8 ± 11.7 kg, height: 182.5 ± 8.9 cm) were recruited to provide a homogeneous participant group. Eligibility criteria were based on the Physical Activity Readiness Questionnaire (PAR-Q) to validate current health status, as well as several exclusion criteria for vibration training [24]. The included participants were not allowed to consume alcohol, caffeine, or nicotine, engage in exercise, use body lotion, undergo therapeutic treatment or massage, or eat a meal within the last two hours before the investigation, as these conditions interfere with the infrared thermography method guidelines [25].

All participants provided written informed consent. This study was performed in accordance with the Declaration of Helsinki and was approved by the local ethics committee ETH-EK (EK 2023-N-28) and the national research committee (GEHBa).

### 2.3. Device to Superimpose Vibration on Stretching

ST+V and ST training conditions could uniquely be performed on the vibration machine TremoFlex (Tremo-Tec GmbH, Oberstdorf, Germany), specifically designed to perform passive stretching training (Supplemental Video S1). The Tremo-Flex consists of a vibrating roll that is adjustable in height up to 150 cm and produces sinusoidal vibration with frequencies between 5 and 30 Hz. The stretching intensity can be continuously increased using the height adjustment with around 5 cm/s until the participant indicates maximal tolerable discomfort.

### 2.4. Infrared Thermography

Skin temperature was measured using an IRT camera, FLIR A500-EST (FLIR Systems, Inc., Wilsonville, OR, USA). IRT images of the legs were performed at 4 time points in a standing position on both the front and back: before (Pre1) and after (Pre2) the pre-test as well as before (Post1) and after (Post2) the post-test. Thermal equilibrium was achieved as the duration between images Pre1 and Pre2 was approximately 15 min.

The camera was placed 0.7 m above the floor, 2.5 m from the measurement position, and at an angle of 90° to the wall, in front of which participants were positioned, to provide a full view of the lower limbs. All thermographic images were analyzed using the FLIR Research Studio Software Version 2.0.0 (FLIR Systems, Inc., Wilsonville, OR, USA). The Regions of Interest (ROI) were set as rectangles of similar amounts of pixels for each participant over the whole leg (end of trousers to foot, pixel: 9859 ± 164) as well as on quadriceps/hamstrings (upper leg area to knee, pixel: 7211 ± 189), knee/popliteal fossa (front: coldest area, back: hottest area, pixel: 1248 ± 2), shin/calf (area of M. Gastrocnemius, pixel: 2041 ± 8), and ankle/heel (area between ankles to heel, pixel: 753 ± 1). The mean temperature was calculated from these ROIs.

Additionally, the room temperature was controlled with a digital temperature and humidity meter (Noklead, Vooco EU, Guangdong, China) and held constant over the course of the intervention.

### 2.5. Myotonometry

The MyotonPRO (Myoton AS, Tallin, Estland) records the damped oscillation induced by five mechanical impulses with an accelerometer and calculates the following parameters from the recorded tissue response [26]: F = Muscle tone (Hz) described as the natural frequency of the acceleration, S = Stiffness (N/m) calculated via the damped natural oscillation response, D = decrement as the shape restoration from deformation, R = relaxation (ms) via the time of the muscle recovery process, and C = creep as gradual tissue elongation under constant stress (Supplemental Figure S1). Despite myotonometry delivering different stiffness values (N/m) compared to shear-wave elastography (kPa), they showed moderate to good correlations [27]. Measurement points were marked with a marker on the muscles Biceps femoris, Semitendinosus, Gastrocnemius lateralis, and Gastrocnemius medialis, as well as on the tendons of Biceps femoris, Semitendinosus, and the Achilles tendon.

### 2.6. Range of Motion Testing via Goniometry

**Figure 2:**
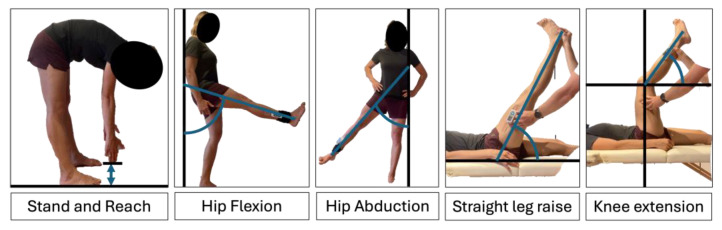
Range of motion testing using goniometry for active hip flexion, active hip abduction, passive straight leg raise, and passive knee extension, as well as the active stand and reach test.

We used a handheld digital Goniometer (EasyAngle, Meloq devices, Stockholm, Sweden) to determine ROM in the following stated tests (Figure 2). An algorithm minimizing disturbing angular components like rotation is incorporated into the digital device.

The Stand and Reach (S&R)**:** The test was performed to assess dorsal chain flexibility. During the test, subjects were instructed to hold both hands next to each other and flex their trunk slowly with straight legs. The maximum distance reached and held for two seconds was used for further data processing.

Hip Flexion (AV): The participant stood with their back on a wall and positioned their feet at a distance of 15 cm from the wall. The foot position was marked with tape. With the back fixed at the wall, the participants were asked to lift their legs upwards as high as possible sagittally.

Hip Abduction (AbD): The participants adopted a sideward standing position with their feet at a distance of 15 cm from the wall. The foot position was marked with tape. With the shoulder fixed at the wall, the participants were instructed to lift their leg with an extended knee joint as high as possible in the frontal plane.

Passive straight leg raise (SLR): Each participant was positioned supine on a massage table, and one leg was fixed with a lashing strap [28]. The other leg was then passively extended by the examiner until the participant reported maximum tolerance.

Passive knee extension test (KE): Participants were positioned supine with one leg fixated while the other hip was flexed to 90 degrees and stabilized. The examiner then passively extended the knee to the end of the range, at which point the knee flexion angle was measured.

### 2.7. Stretching Protocol

**Figure 3:**
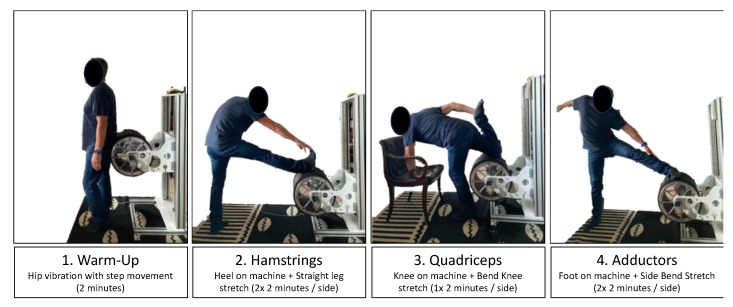
Stretching positions performed in ST+V and ST. Each position was held for 2 × 2 min on each side with (ST+V) or without (ST) superimposed vibration. Additional information is provided in the Supplementary Video S1.

ST+V and ST contained supervised passive stretching with three exercises targeting hamstrings, quadriceps, and adductors for two repetitions each of 2 min duration (quadriceps: 1 × 2 min) on each side (Figure 3). The three exercises with two repetitions on the left and right sides, plus a 2 min vibration warm-up, took 22 min in total. ST+V and ST training conditions performed all exercises on the vibration machine TremoFlex (Tremo-Tec GmbH, Oberstdorf, Germany), while the vibration was exclusively applied under the combined condition (ST+V). For the hamstring and adductor exercises, the participants lifted their foot on the vibration roll and kept their knee joint straight (assisted and controlled by the therapist), while for the hamstring exercise, the knee was placed on the vibration roll, and the foot was pressed to the buttocks (assisted by the therapist). The stretching intensity was continuously increased using the height adjustment until the participant indicated maximal tolerable discomfort. Only ST+V stretched with superimposed vibration using a fixed frequency of 20 Hz with a corresponding amplitude of 7 mm and calculated acceleration of 110.5 m/s^2^ or 11.3 g [29]. The stretching procedure can be watched in Video S1 in the Supplemental Material.

During the stretch training, we noted the height the athletes reached in every flexibility exercise. Exercises were performed barefoot.

### 2.8. Statistical Analysis

All data were analyzed using SPSS Version 28.1 statistical software (SPSS Inc., Chicago, IL, USA). Data are presented as mean ± standard deviation (SD). Normal distribution was checked using the Shapiro–Wilk test. Reliability was determined and is provided with an intraclass-correlation coefficient (ICC) and 95% confidence intervals (CI) for the aforementioned tests (Supplemental Table S1). Also, 95% CI for ICC was interpreted considering the guidelines by Koo and Li [30]: poor reliability ≤ 0.5, moderate reliability = 0.5–0.75, good reliability ≥ 0.75–0.9, excellent reliability ≥ 0.9.

Moreover, homogeneity of variances was performed by Levene’s test. A one-way analysis of variance (ANOVA) was used to confirm no significant condition differences in the pre-test values.

For calculation of an overall within-subject effect, we used a two-way ANOVA with repeated measurements with the within-subject variable “Time” (2 levels: Pre, Post) and the between-subject factor “Intervention” (3 groups: ST+V, SS, CG), followed by a post hoc analysis with Bonferroni adjustment.

The level of significance was set at *p* < 0.05. Effect sizes are presented as Eta squares (η^2^) and categorized as follows: small effect η^2^ < 0.06, medium effect η^2^ = 0.06–0.14, high effect η^2^ > 0.14 [31]. Additionally, effect sizes are reported with Cohen’s d [31] and categorized as small effects (d < 0.5), medium effects (d = 0.5–0.8), and high effects (d > 0.8).

## 3. Results

Reliability analysis revealed ICCs between 0.72–0.99 (for detailed results, see Supplemental Table S1), with normal distribution assumed for almost all (>90% of parameters) (*p* = 0.065–0.999). For all parameters, no group differences were obtained in pre-testing (*p* = 0.100–0.967). Three measurements could not be conducted due to the absence of participants.

### 3.1. Range of Motion

**Figure 4:**
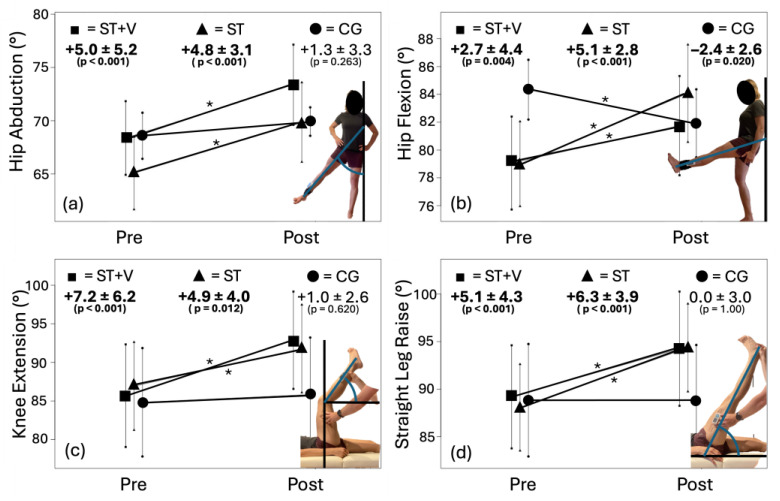
Range of motion (ROM) measurements before (pre) and after (post) the interventions of stretching with vibration (ST+V—■), stretching alone (ST—▲), and control (CG—●) for (**a**) hip abduction, (**b**) hip flexion, (**c**) passive knee extension, and (**d**) passive straight leg raise test with significant pre-post differences (*).

There were large-magnitude ROM increases (η^2^ = 0.219–0.759, *p* < 0.001–0.002) (Supplemental Table S2). Large-magnitude Time X Intervention interactions (η^2^ = 0.145–0.444, *p* < 0.001–0.047) indicated between-group differences in the responses. Post hoc testing revealed ROM increases overall testing conditions (*p* < 0.001–0.012, η^2^ = 0.198–0.717), showing that ST+V and ST increased their flexibility significantly, while no changes were observed in the CG (Figure 4). However, there were no significant differences in ROM increases between the ST+V and ST intervention conditions (*p* = 0.202–0.999).

### 3.2. Myotonometry

Myotonometry (Supplemental Table S3) showed significant main effects for test time points in semitendinosus tendon decrement (η^2^ = 0.095, *p* = 0.049) and for the gastrocnemius lateralis stiffness (η^2^ = 0.120, *p* = 0.026), indicating in all conditions a significant pre-post change. The “Time X Intervention” interaction reached the level of significance for gastrocnemius lateralis decrement (η^2^ = 0.160, *p* = 0.033) and the stiffness of the Achilles tendon (η^2^ = 0.036, *p* = 0.036), while in post hoc testing only CG demonstrated significant decrease and increase, respectively.

### 3.3. Skin Temperature

Room temperature was held constant, as there were only minor differences over the course of a measurement (Day 1: +0.03 °C, Day 2: +0.05 °C, Day 3: −0.04 °C) without a significant difference within days (*p* = 0.75).

**Figure 5:**
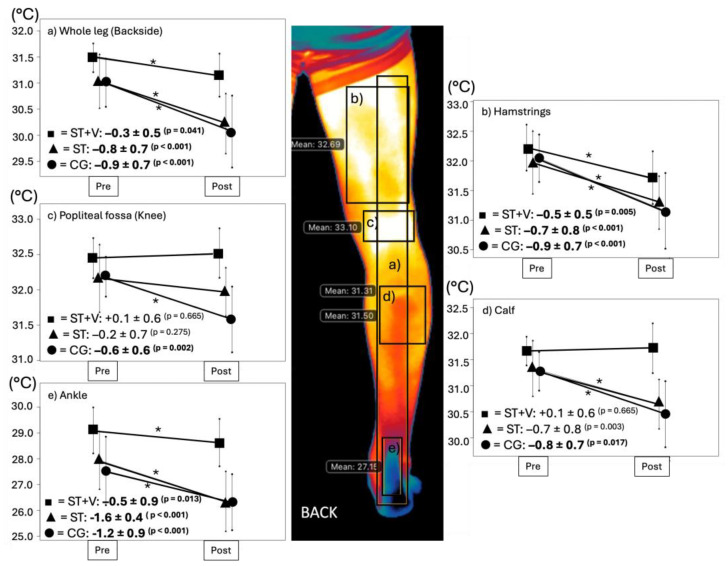
Leg skin temperature (°C) backside from before (pre) to after (post) the interventions stretching with vibration (ST+V—■), stretching alone (ST—▲), and control (CG—●) for the regions of interest (**a**) whole leg, (**b**) quadriceps, (**c**) knee/popliteal fossa, (**d**) calf muscle, and (**e**) ankle/heel with significant pre-post differences (*).

The skin temperature of the investigated leg areas (Figure 5) reported overall significant decreases over the course of the study with small to high effect sizes (η^2^ = 0.053–0.656, *p* < 0.001–0.147), but differentiated between interventions demonstrated by the interaction effects (η^2^ = 0.027–0.273, *p* = 0.002–0.587). The interaction effect revealed significant group differences for the ROI ankle frontside (*p* = 0.041), whole leg backside (*p* = 0.032), knee/popliteal fossa backside (*p* = 0.031), calf backside (*p* = 0.011), and heel backside (*p* = 0.002). Post hoc analysis showed no temperature change in ST+V but significant temperature decreases in ST (*p* = 0.002–0.047, d = 0.7–1.2) and CG (*p* = 0.020–0.047, d = 0.7–0.8) in the backside spots (Supplemental Table S4).

## 4. Discussion

In accordance with the initially stated hypothesis, the stretching interventions (ST) showed significant ROM improvements. However, in contrast to our assumption, superimposed vibration (ST+V) did not provide additional benefits compared to the stretching alone group. Only for the skin temperature did the additional vibration condition show an effect, as in the other two groups (S and CG); the skin temperature dropped from pre- to post-testing, while the ST+V group could hold their temperature. These temperature effects were, however, not associated with additional flexibility gains, nor were viscoelastic parameters as accessed by myotonometry.

In accordance with previous literature, we confirmed that stretching acutely enhanced flexibility [15,32,33]. Nevertheless, previous studies indicated that additional vibration effects could provide further benefits [34,35,36,37,38,39,40]. Since these studies compared the effect of vibration versus a passive control, it cannot be ruled out that these results can be attributed to general warm-up effects [16]. Therefore, an active control condition, using the stand-alone condition (in our case, the stretching alone group), is urgently needed to reasonably investigate whether vibration provides an additional benefit. Although some literature exists that superimposes vibration to stretching, study results are controversial. Some authors found additional effects [18,41,42,43,44], while others did not [17,19,20,21,45,46,47]. Interestingly, none out of 46 reviewed acute studies performed blinding of assessors, according to a recent meta-analysis [15]. As a consequence, and due to the conflicting evidence, to shed light on the potential positive effects of superimposed vibration stimuli, it seems necessary to explore underlying mechanisms to find potential moderators of vibration effects.

### 4.1. Underlying Mechanisms of ROM Increases: Stiffness Effects

Stiffness parameters are well-known to be affected by stretching [8,48] and can be considered moderately related to ROM values [49]. In contrast to the ROM effects of vibration [14], local vibration itself seems not to affect passive resistive torque during passive knee extension [40] or muscle stiffness measured by the shear elastic modulus of passive quadriceps muscles [50]. Interestingly, Warneke et al. [16] screened the available literature and showed no stiffness difference between stretching and alternatives (including vibration) in acute settings. Concerning the combination of stretching with vibration, we are only aware of one study [17] that measured stiffness with a ratio between passive torque variation and ROM. While stretching alone led to decreased stiffness, Rodrigues et al. [17] found no effect of vibration or the combination of stretching with vibration. Therefore, our results are in accordance with the literature, as stretching alone seemed to affect stiffness, and vibration did not provide additional benefits. As mentioned for ROM increases as well, these changes could be the result of general warm-up effects [16].

Further, as the pain threshold also determines ROM [51], the highest stretch subjects can tolerate is measured with the passive peak torque at the end ROM [4]. Vibration was proposed to alter pain sensation [52], but Rodrigues et al. [17] also found no difference in passive peak torque in agreement with us finding no difference in ROM. Therefore, more research concerning underlying mechanisms is warranted.

### 4.2. Temperature Effects

In contrast to de Oliveira et al. [53], our study showed passive stretching did not cause an increase in skin temperature. This might be due to their better methodology, where participants did not have to change the room between intervention and measurements. In our study, there was a drop in skin temperature in the stretching-alone group but also in the control group, while when vibration was added, this temperature drop could be diminished. However, some limitations must be considered when drawing conclusions. On the one hand, skin temperature might not be equal to muscle temperature; therefore, the influence on muscle properties cannot be assessed. On the other hand, Schleip [54] indicated that the skin and myofascial are permeated by sensory receptors, which are sensible to skin stimulation, tangential forces, or stretch [55]. These were described as potentially polysynaptically linked to motoneurons [56,57]. The exteroceptive reflexes could potentially contribute to relaxation and decreased muscle tone, which could, in turn, affect ROM. Nevertheless, even though vibrational training could also contribute to exteroceptive reflexes, Behm et al. [58] stated that only large amplitude stretches have the potential to influence these mechanisms, with a questionable influence on ROM parameters. Irrespective of imposed effects, increased skin temperature and speculated vibration-specific effects were ineffective in inducing superior ROM/stiffness improvements compared to stretching alone. Potential usage of the skin temperature effect induced by vibration might be beneficial for the delayed onset of muscle soreness, as conducted before [59].

### 4.3. Limitations

Though the small participant number is a big limitation of the study, this was thought of as a necessity to increase homogeneity by incorporating only youth elite athletes from the same team with a similar training status and the same training plan (to increase intersubject homogeneity). The athletes provided already high ROM due to consistent flexibility routines, e.g., regular supervised stretching training. Therefore, we assumed the potential benefits of adding vibration to further increase the already high flexibility status. But as it seems, the passive stretching training at the vibration device itself also provided a sufficient stimulus to increase the range of motion in several tests. As this study population was rather specific, whether our findings are predicative for other subject groups remains to be determined. With our participants, we achieved a statistical power of 0.76–0.99 for ROM, while viscoelastic parameters measured with myotonometry peaked at 0.80 (assumed correlation: 0.3) using G*Power (Version 3.1.9.6) [60]. The incorporated type 2 error questions the accuracy of our results, especially for stiffness measures, while ROM seems rather stable. Nevertheless, this demands further research with a higher number of participants.

Possible moderators like stiffness and skin temperature were not related to the changes in ROM. However, in contrast to previous similar literature, viscoelastic properties were measured here via myotonometry. Even though it is a frequently performed testing procedure, its reproducibility and thus reliability and validity is limited. In accordance with ICC = 0.716–0.976 found in our study, future research should implement more reliable and valid stiffness measurements (e.g., elastography, dynamometry). Although the considered moderators were not related to the main outcome, future research should investigate if more constitutions (body composition, BMI) or sex-specific parameters show a significant influence on ROM adaptations induced by vibration. Furthermore, it is possible that static stretching as a passive intervention was not the correct stimulus. Future research should, therefore, investigate different exercise types and test for further moderators and underlying mechanisms that were not considered in this article.

Though very precise, the nature of skin temperature drift over the course of the study makes an interpretation of the results complex, as the clinical relevance of skin temperature differences seems negligible when smaller than 0.5 °C [61].

### 4.4. Practical Relevance

Preparing for subsequent physical activity is of high relevance to increase performance. Therefore, activities to optimize flexibility and maximize active ROM are urgently warranted. While stretching is one of the most common activities to improve ROM acutely and chronically [1,32], imposing additional stimulation was discussed to emphasize responses and accumulate effects [15,16]. Although it was hypothesized that vibration improved ROM or reduced stiffness, no such additional benefits were observed in this study, questioning the results of previous articles that suggested additional effects. Since no inter-intervention effects were observed, no additional benefits for practically relevant outcomes can be highlighted, supporting results of a recently published meta-analysis that indicated that there might be no specific effects of different interventions on acute ROM, as acute ROM improvements might be the result of general warm-up effects. Therefore, our data suggest that there is no need to perform additional vibration if the aim is to improve ROM and decrease stiffness acutely.

## 5. Conclusions

Even though the use of vibration devices is in vogue, our results showed that an acute bout of stretching superposed with vibration did not provide an additional benefit compared to stretching alone, at least for young athletes. However, in comparison to most other acute vibration studies, our pilot study focused on a homogenous, though small, sample size and lifted study quality by conducting a blinded data collection. Still, further research, including a larger sample size as well as chronic interventions comparing vibration training with a sham condition, is required to draw a more conclusive picture of vibration effects, as potential acute effects could also be induced by warm-up effects. Chronic effects might induce higher ROM in recreational or sedentary populations due to enhanced activity in general, which might be reached by performing any activity. Therefore, when aiming to investigate specific intervention effects, it is mandatory to include a comparison sham group.

## Data Availability

Data can be openly accessed in the Supplementary Material.

## Supplementary Materials

The following supporting information can be downloaded at: https://www.mdpi.com/article/10.3390/jfmk10010017/s1, Figure S1. Myotonometry measurement procedure, parameters, and used measurement points: MyotonPRO records damped oscillations induced by mechanical impulses (preload: 0.18 N + impulse force: 0.42 N, depth: <20 mm, impulse time: 0.15 ms, registration: 385 ms, signal processing: 150 ms, parameter computation: 50 ms) with an accelerometer, averages five executed oscillations (frequency interval: 0.8 s between single measurements), and calculates parameters characterizing intrinsic tension of biological soft tissue (frequency, computed by Fast Fourier Transformation from the signal spectrum fmax), resistance of the tissue to deformation (stiffness), damping of tissue oscillation (logarithmic decrement), tissue’s recovery time from displacement (relaxation), and gradual elongation of tissue over time when placed under constant tensile stress (creep) with the respective formulas; extracted from MyotonPRO user manual (https://www.myoton.com/UserFiles/Updates/MyotonPRO_User_Manual.pdf, URL accessed on 31 October 2024). Table S1. Intraclass Correlation Coefficients (ICC) with 95% Confidence Intervals (95%-CI). Table S2. Descriptive statistics and results of two-way ANOVA for a range of motion measured by the stand-and-reach test (S&R), active hip anteversion (AV), active hip abduction (AbD), passive straight leg raise (SLR), and knee-extension test (KE). Table S3. Descriptive statistics and results of two-way ANOVA for viscoelastic characteristics of myotonometry for groups Stretching + Vibration (ST+V), Stretching alone (ST), and Control group (CG). Table S4. Descriptive statistics and results of two-way ANOVA for leg skin temperature (°C) of ROI Leg (Front/Back), Quadriceps/Hamstrings, Knee/Popliteal fossa, Shin/Calf, Ankle/Heel with respective amounts of pixels (Px) for groups Stretching + Vibration (ST+V), Stretching alone (ST), and Control group (CG). Video S1. Stretching positions performed in ST+V and ST. Each position was held for 2 × 2 min on each side with (ST+V) or without (ST) superimposed vibration.
