# Supplementary material for: Acute Effects of Passive Stretching with and Without Vibration on Hip Range of Motion, Temperature, and Stiffness Parameters in Male Elite Athletes
Source: J Funct Morphol Kinesiol. 2025 Jan 2;10(1):17. doi: 10.3390/jfmk10010017 (PMC11755640; doi:10.3390/jfmk10010017)
Supplement: Supplementary file 1 [file jfmk-10-00017-s001.zip › jfmk-3366211-supplementary.pdf]

SUPPLEMENTAL MATERIAL

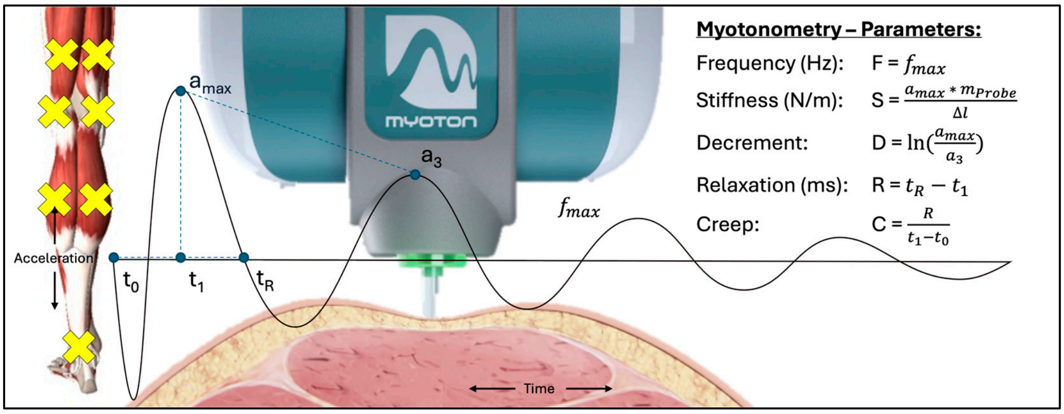

Figure S1. Myotonometry measurement procedure, parameters and used measurement points: MyotonPRO records damped oscillations induced by mechanical impulses (preload: 0.18N + impulse force: 0.42N, depth: <20mm, impulse time: 0.15ms, registration: 385ms, signal processing: 150ms, parameter computation: 50ms) with an accelerometer, averages five executed oscillations (frequency interval: 0.8s between single measurements) and calculates parameters characterizing intrinsic tension of biological soft tissue (F = frequency, computed by Fast-Fourier Transformation from the signal spectrum f_{max}), resistance of the tissue to deformation (S = stiffness), damping of tissue oscillation (D = logarithmic decrement), tissue's recovery time from displacement (R = relaxation), and gradual elongation of tissue over time when placed under constant tensile stress (C = creep) with the respective formulas; Acceleration in milliG, Time in milli seconds (ms); a_{max}: maximum displacement, a_3: maximum displacement of the second oscillation period m_{probe}: mass of the measurement system, Δl: maximum displacement of the tissue, t_0: start of the mechanical impulse, t_1: timepoint of maximum displacement, t_R: timepoint of tissue recovery from being deformed; extracted from MyotonPRO user manual (https://www.myoton.com/UserFiles/Updates/MyotonPRO_User_Manual.pdf)

Table S1. Intraclass Correlation Coefficients (ICC) with 95% Confidence Intervals (95%-CI)

Parameter	ICC (95%-CI)
Flexibility	0.991 (0.986 – 0.995)
Frequency	0.957 (0.939 – 0.971)
Stiffness	0.976 (0.966 – 0.984)
Decrement	0.716 (0.592 – 0.807)
Relaxation time	0.971 (0.958 – 0.980)
Creep	0.975 (0.964 – 0.983)
Temperature	0.925 (0.899 – 0.946)

Table S2. Descriptive statistics and results of two-way ANOVA for Range of motion measured by stand-&-reach test (S&R), active hip anteversion (AV), active hip abduction (AbD), passive straight leg raise (SLR), and knee-extension test (KE).

		n	Pre (cm)	Post (cm)	Difference (%)	Time	Time * Int
S&R	ST+V	8	9.3 ± 6.3	11.9 ± 6.0	**+ 27.9** (p < .001)	**$p < .001$**	**$p = .036$**
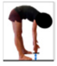	SS	7	7.4 ± 3.4	9.3 ± 4.0	**+ 25.7** (p < .001)	**$F_{18, 1}$ = 56.755**	**$F_{18, 2}$ = 4.022**
	CG	6	7.8 ± 4.7	8.8 ± 4.7	+ 12.8 (p = .051) *	**η^2 = .759**	**η^2 = .309**
		**n**	**Pre (°)**	**Post (°)**	**Difference (%)**	**Time**	**Time * Int**
AV	ST+V	8	79.1 ± 6.8	81.8 ± 7.2	**+ 3.3** (p = .004)	**$p = .002$**	**$p < .001$**
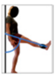	SS	7	79.0 ± 5.8	84.1 ± 6.7	**+ 6.5** (p < .001)	**$F_{39, 1}$ = 10.964**	**$F_{39, 2}$ = 15.594**
	CG	6	84.3 ± 3.8	81.9 ± 4.4	**- 2.8** (p = .020) *○	**η^2 = .219**	**η^2 = .444**
AbD	ST+V	8	68.4 ± 7.1	73.4 ± 7.7	**+ 7.3** (p < .001)	**$p < .001$**	**$p = .047$**
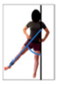	SS	7	65.1 ± 6.5	69.9 ± 7.1	**+ 7.4** (p < .001)	**$F_{39, 1}$ = 34.406**	**$F_{39, 2}$ = 3.307**
	CG	6	68.6 ± 3.9	69.9 ± 2.4	+ 1.9 (p = .263)	**η^2 = .469**	**η^2 = .145**
SLR	ST+V	8	89.2 ± 11.1	94.3 ± 12.3	**+ 5.7** (p < .001)	**$p < .001$**	**$p < .001$**
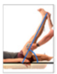	SS	7	88.1 ± 8.7	94.3 ± 8.8	**+ 7.0** (p < .001)	**$F_{39, 1}$ = 41.003**	**$F_{39, 2}$ = 9.794**
	CG	6	88.8 ± 10.4	88.8 ± 10.3	± 0.0 (p = 1.00) *○	**η^2 = .513**	**η^2 = .222**
KE	ST+V	8	85.7 ± 14.4	92.9 ± 12.9	**+ 8.4** (p < .001)	**$p < .001$**	**$p = .005$**
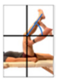	SS	7	86.9 ± 10.9	91.8 ± 10.8	**+ 5.6** (p = .012)	**$F_{39, 1}$ = 35.594**	**$F_{39, 2}$ = 5.987**
	CG	6	84.8 ± 12.4	85.8 ± 13.1	+ 1.2 (p = .620) *	**η^2 = .477**	**η^2 = .235**

Table S3. Descriptive statistics and results of two-way ANOVA for viscoelastic characteristics of myotonometry for groups Stretching + Vibration (ST+V), Stretching alone (ST), and Control group (CG).

	Group			Time effect			Time * Intervention		
**M.BF**	**ST+V (n=8)**	**ST (n=7)**	**CG (n=6)**	**p**	**F**	**η²**	**p**	**F**	**η²**
**F**	-0.11 ± 0.3	0.20 ± 0.5	0.24 ± 0.7	.173	1.925	.047	.136	2.103	.097
**S**	-1.68 ± 10.0	5.60 ± 15.5	**8.70 ± 18.0**	.069	3.495	.082	.158	1.936	.090
**D**	0.00 ± 0.1	0.01 ± 0.1	-0.02 ± 0.1	.814	.056	.001	.312	1.199	.058
**R**	0.15 ± 0.5	-0.37 ± 0.9	-0.10 ± 0.9	.367	.832	.021	.192	1.724	.081
**C**	0.01 ± 0.0	*-0.02 ± 1.0*	0.00 ± 0.0	.686	.165	.004	.117	2.268	.104
**M.ST**	**ST+V (n=8)**	**ST (n=7)**	**CG (n=6)**	**p**	**F**	**η²**	**p**	**F**	**η²**
**F**	-0.17 ± 0.4	-0.05 ± 0.4	0.22 ± 0.5	.975	.001	.000	.081	2.680	.121
**S**	2.28 ± 12.2	0.56 ± 31.9	5.42 ± 8.6	.146	2.200	.053	.584	.545	.027
**D**	0.00 ± 0.1	0.00 ± 0.0	0.01 ± 0.0	.682	.171	.004	.961	.040	.002
**R**	-0.08 ± 0.7	0.07 ± 0.8	-0.16 ± 0.5	.604	.273	.007	.694	.369	.019
**C**	-0.01 ± 0.0	0.01 ± 0.0	-0.01 ± 0.0	.813	.057	.001	.688	.378	.019
**T.BF**	**ST+V (n=8)**	**ST (n=7)**	**CG (n=6)**	**p**	**F**	**η²**	**p**	**F**	**η²**
**F**	0.02 ± 0.7	0.01 ± 0.8	0.25 ± 0.4	.396	.736	.019	.590	.535	.027
**S**	3.39 ± 28.3	2.39 ± 23.9	7.70 ± 11.5	.218	1.565	.039	.827	.190	.010
**D**	0.00 ± 0.1	0.01 ± 0.0	-0.01 ± 0.1	.860	.032	.001	.458	.737	.036
**R**	-0.06 ± 0.8	-0.05 ± 0.8	-0.19 ± 0.4	.360	.857	.022	.849	.164	.008
**C**	-0.00 ± 0.0	0.00 ± 0.0	-0.01 ± 0.0	.751	.102	.003	.865	.146	.007
**T.ST**	**ST+V (n=8)**	**ST (n=7)**	**CG (n=6)**	**p**	**F**	**η²**	**p**	**F**	**η²**
**F**	0.33 ± 0.8	0.08 ± 0.5	0.05 ± 0.6	.840	.041	.001	.920	.084	.004
**S**	7.52 ± 48.4	-1.51 ± 58.4	2.98 ± 36.8	.697	.153	.004	.882	.126	.006
**D**	**-0.06 ± 0.1**	0.01 ± 0.1	-0.06 ± 0.1	**.049**	**4.115**	**.095**	.173	1.836	.086
**R**	0.06 ± 1.3	0.34 ± 1.6	-0.09 ± 1.1	.622	.247	.006	.696	.366	.018
**C**	0.00 ± 0.1	0.02 ± 0.1	-0.00 ± 0.1	.541	.381	.010	.676	.396	.020
**M.GL**	**ST+V (n=8)**	**ST (n=7)**	**CG (n=6)**	**p**	**F**	**η²**	**p**	**F**	**η²**
**F**	0.10 ± 0.6	-0.07 ± 0.5	-0.27 ± 0.8	.137	2.311	.056	.431	.861	.042
**S**	*5.94 ± 12.3*	*6.32 ± 13.3*	1.13 ± 11.7	**.026**	**5.334**	**.120**	.507	.692	.034
**D**	0.01 ± 0.1	0.04 ± 0.1	**-0.05 ± 0.1°**	.829	.048	.001	**.033**	**3.719**	**.160**
**R**	-0.28 ± 0.7	-0.20 ± 0.8	-0.06 ± 1.0	.155	2.106	.051	.776	.256	.013
**C**	-0.01 ± 0.0	-0.01 ± 0.1	-0.00 ± 0.1	.337	.945	.024	.795	.231	.012
**M.GM**	**ST+V (n=8)**	**ST (n=7)**	**CG (n=6)**	**p**	**F**	**η²**	**p**	**F**	**η²**
**F**	0.10 ± 0.6	-0.07 ± 0.5	-0.27 ± 0.8	.424	.652	.016	.311	1.203	0.58
**S**	3.65 ± 8.8	-1.94 ± 7.9	2.02 ± 10.4	.380	.790	.020	.237	1.495	.071
**D**	-0.01 ± 0.1	0.03 ± 0.1	-0.02 ± 0.1	.841	.041	.001	.276	1.330	.064
**R**	-0.24 ± 0.7	-0.27 ± 0.6	-0.15 ± 0.7	.686	.166	.004	.072	2.812	.126
**C**	-0.01 ± 0.0	0.02 ± 0.0	-0.00 ± 0.0	.784	.076	.002	.106	2.382	.109
**AT**	**ST+V (n=8)**	**ST (n=7)**	**CG (n=6)**	**p**	**F**	**η²**	**p**	**F**	**η²**
**F**	-0.16 ± 1.1	-0.24 ± 2.1	0.75 ± 1.7	.647	.213	.005	.247	1.448	.069
**S**	-9.21 ± 30.9	-5.93 ± 66.5	**36.0 ± 38.6**	.354	.881	.022	**.036**	**3.626**	**.036**
**D**	0.07 ± 0.2	0.01 ± 0.2	-0.07 ± 0.2	.829	.047	.001	.119	2.245	.103
**R**	0.11 ± 0.4	0.18 ± 1.0	-0.35 ± 0.6	.849	.037	.001	.123	2.213	.102
**C**	0.00 ± 0.0	0.01 ± 0.1	-0.02 ± 0.0	.839	.042	.001	.159	1.928	.090

M.BF = biceps femoris muscle, M.ST = semitendinosus muscle, T.BF = biceps femoris tendon, T.ST = semitendinosus tendon, M.GL = gastrocnemius lateralis, M.GM = gastrocnemius medialis, AT = achilles tendon

F = frequency (Hz), S = dynamic stiffness (N/m), D = logarithmic decrement, R = relaxation (ms), C = creep.

Group differences from Pre to Post are represented as mean ± standard deviation.

Time effect // Post-hoc Bonferroni: Bold values indicate a significant difference from Pre to Post of the respective group.

Time * Intervention effect // Post-hoc Bonferroni: Asterisks (*) illustrate a significant ($p < .05$) difference of the respective group from the vibration group. The degree sign (°) illustrates a significant ($p < .05$) difference of the respective group from the stretching group.

Table S4. Descriptive statistics and results of two-way ANOVA for leg skin temperature (°C) of ROI Leg (Front/Back), Quadriceps/Hamstrings, Knee/Popliteal fossa, Shin/Calf, Ankle/Heel with respective amount of Pixels (Px) for groups Stretching + Vibration (ST+V), Stretching alone (ST), and Control group (CG).

**Temperature**	**Front**	**n**	**Pre** (°C)	**Post** (°C)	**Difference (°C)**	**Time effect**	**Time * Int**
**Leg**	ST+V	8	31.4 ± 0.4	31.0 ± 0.8	**- 0.4 ± 0.6** (p = .019)	**p < .001**	p = .253
Front	ST	7	30.8 ± 1.0	30.2 ± 0.9	**- 0.6 ± 0.9** (p = .003)	**$F_{39,1}$ = 33.993**	$F_{39,2}$ = 1.426
Px: 9859 ± 164	CG	6	30.7 ± 0.7	29.9 ± 1.1	**- 0.8 ± 0.5** (p < .001)	**η^2 = .466**	η^2 = .068
**Quadriceps**	ST+V	8	32.5 ± 0.7	31.8 ± 1.0	**- 0.7 ± 0.7** (p = .002)	**p < .001**	p = .359
Front	ST	7	31.6 ± 1.2	31.0 ± 1.0	**- 0.6 ± 1.1** (p = .009)	**$F_{39,1}$ = 36.329**	$F_{39,2}$ = 1.052
Px: 7211 ± 189	CG	6	31.9 ± 0.9	30.8 ± 1.1	**- 1.1 ± 0.6** (p < .001)	**η^2 = .482**	η^2 = .051
**Knee**	ST+V	8	31.0 ± 0.9	30.4 ± 0.9	**- 0.6 ± 0.7** (p = .009)	**p < .001**	p = .587
Front	ST	7	30.1 ± 1.8	29.6 ± 1.3	**- 0.5 ± 1.1** (p = .037)	**$F_{39,1}$ = 23.144**	$F_{39,2}$ = 0.540
Px: 1248 ± 2	CG	6	29.8 ± 0.8	29.1 ± 1.3	**- 0.8 ± 0.6** (p = .002)	**η^2 = .372**	η^2 = .027
**Shin**	ST+V	8	31.9 ± 0.5	32.0 ± 0.6	+ 0.1 ± 0.5 (p = .602)	p = .147	p = .090
Front	ST	7	31.7 ± 0.8	31.6 ± 0.6	- 0.1 ± 0.9 (p = .707)	$F_{39,1}$ = 2.189	$F_{39,2}$ = 2.565
Px: 2041 ± 7	CG	6	31.7 ± 0.6	31.2 ± 0.9	**- 0.5 ± 0.5** (p = .017)	η^2 = .053	η^2 = .116
**Ankle**	ST+V	8	30.8 ± 1.4	30.4 ± 1.1	**- 0.4 ± 0.6** (p = .013) °	**p < .001**	**p = .041**
Front	ST	7	30.0 ± 1.5	29.0 ± 1.5	**- 1.0 ± 0.3** (p < .001) *	**$F_{39,1}$ = 56.197**	**$F_{39,2}$ = 3.460**
Px: 753 ± 1	CG	6	29.4 ± 2.5	28.6 ± 2.3	**- 0.8 ± 0.8** (p < .001)	**η^2 = .590**	**η^2 = .151**
**Temperature**	**Back**	**n**	**Pre** (°C)	**Post** (°C)	**Difference (°C)**	**Time effect**	**Time * Int**
**Leg**	ST+V	8	31.5 ± 0.6	31.2 ± 0.8	**- 0.3 ± 0.5** (p = .041)	**p < .001**	**p = .032**
Back	ST	7	31.0 ± 1.0	30.2 ± 1.1	**- 0.8 ± 0.7** (p < .001)	**$F_{39,1}$ = 50.443**	**$F_{39,2}$ = 3.753**
Px: 9859 ± 164	CG	6	31.0 ± 0.7	30.1± 1.2	**- 0.9 ± 0.7** (p < .001) *	**η^2 = .564**	**η^2 = .161**
**Hamstrings**	ST+V	8	32.2 ± 0.8	31.8 ± 1.0	**- 0.5 ± 0.5** (p = .005)	**p < .001**	p .352
Back	ST	7	32.0 ± 1.0	31.3 ± 1.0	**- 0.7 ± 0.8** (p < .001)	**$F_{39,1}$ = 42.596**	$F_{39,2}$ = 1.072
Px: 7211 ± 189	CG	6	32.0 ± 0.7	31.1 ± 1.1	**- 0.9 ± 0.7** (p < .001)	**η^2 = .522**	η^2 = .052
**Popliteal fossa**	ST+V	8	32.4 ± 0.6	32.5 ± 0.7	+ 0.1 ± 0.6 (p = .665)	**p = .021**	**p = .031**
Back	ST	7	32.2 ± 0.9	32.0 ± 0.7	- 0.2 ± 0.7 (p = .275)	**$F_{39,1}$ = 5.806**	**$F_{39,2}$ = 5.806**
Px: 1248 ± 2	CG	6	32.2 ± 0.5	31.6 ± 0.8	**- 0.6 ± 0.6** (p = .002) *	**η^2 = .130**	**η^2 = .163**
**Calf**	ST+V	8	31.6 ± 0.6	31.7 ± 1.0	+ 0.1 ± 0.8 (p = .787) °	**p < .001**	**p = .011**
Back	ST	7	31.3 ± 1.0	30.6 ± 0.9	**- 0.7 ± 0.8** (p = .003) *	**$F_{39,1}$ = 15.196**	**$F_{39,2}$ = 5.086**
Px: 2041 ± 7	CG	6	31.3 ± 0.7	30.5 ± 1.1	**- 0.8 ± 0.7** (p = .017) *	**η^2 = .280**	**η^2 = .207**
**Heel**	ST+V	8	29.1 ± 1.8	28.6 ± 1.8	**- 0.5 ± 0.9** (p = .013) °	**p < .001**	**p = .002**
Back	ST	7	27.9 ± 2.1	26.3 ± 2.2	**- 1.6 ± 0.4** (p < .001) *	**$F_{39,1}$ = 74.399**	**$F_{39,2}$ = 7.320**
Px: 753 ± 1	CG	6	27.5 ± 2.3	26.3 ± 1.9	**- 1.2 ± 0.9** (p < .001)	**η^2 = .656**	**η^2 = .273**

Video S1. Stretching positions performed in ST+V and ST. Each position was held for 2x2 minutes on each side with (ST+V) or without (ST) superimposed vibration.
